# Supplementary material for: Short-Term Soy Bread Intervention Leads to a Dose-Response Increase in Urinary Isoflavone Metabolites and Satiety in Chronic Pancreatitis
Source: Foods. 2023 Apr 24;12(9):1762. doi: 10.3390/foods12091762 (PMC10178207; doi:10.3390/foods12091762)
Supplement: Supplementary file 1 [file foods-12-01762-s001.zip › foods-2284445-supplementary.pdf]

Supplementary Results Figure S1

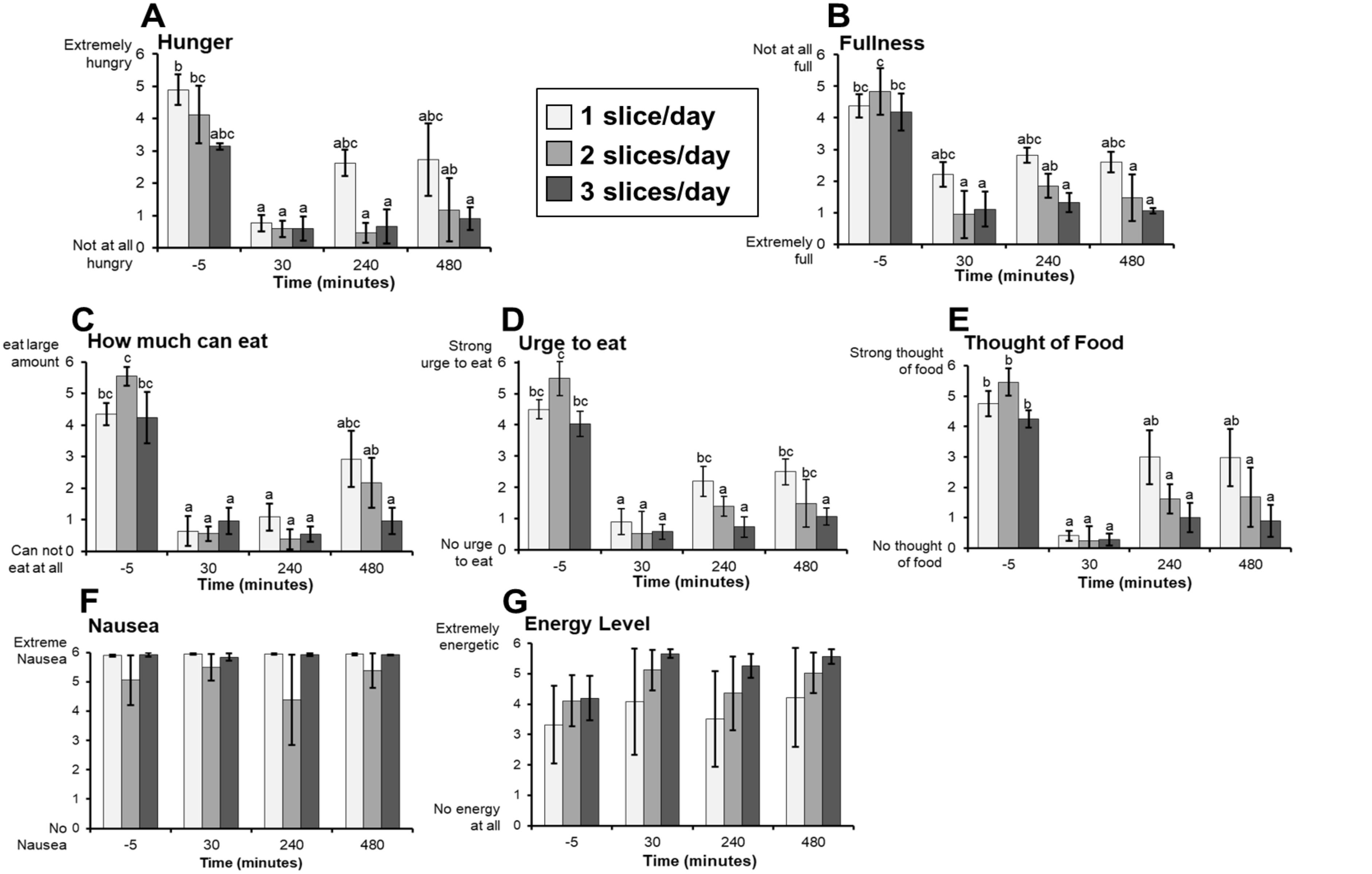

Figure S1. VAS scores were used to assess soy bread satiety. A to G, VAS scores were used to assess satiety over 8 hours and across three doses. Satiety was represented as bar graphs (mean ± SD). Superscript letters represent the mean separation using one way ANOVA analysis and Tukey's post hoc test when significant differences ($p \leq 0.05$) were observed. *Significant differences ($p \leq 0.05$) between soy bread and ideal bread using a paired t-test.
